# Managing Epileptic Seizures by Controlling the Brain Driver Nodes: A Complex Network View

**DOI:** 10.3389/fbioe.2013.00021

**Published:** 2013-12-12

**Authors:** Fatemeh Bakouie, Shahriar Gharibzadeh, Farzad Towhidkhah

**Affiliations:** ^1^Neural and Cognitive Sciences Laboratory, Biomedical Engineering Faculty, Amirkabir University of Technology, Tehran, Iran; ^2^Cybernetics and Modeling of Biological Systems Laboratory, Biomedical Engineering Faculty, Amirkabir University of Technology, Tehran, Iran

**Keywords:** epileptic seizure, synchronization, brain networks, controllability, driver nodes

The brain is a complex biological organization. In its hierarchy, different components, from neurons to functional cognitive circuits are interacting with each other. As a result of cooperation between neurons in the lower levels of this hierarchy, high level cognitive functions emerge (Stam and Reijneveld, 2007). In order to uncover the complexity of these higher functions, understanding the interaction rules in the lower level may be useful. In this level, there are lots of components which connect to each other (with a special structure) and exchange their information (in a specific manner). In this regard, complex network approach will be an influential way to study brain organization. The brain connectivity structure is suggested as a basis for emergence of its complex functions (Rubinov et al., 2009). For example, brain network analysis shows that its connectivity has the “small-worldness” feature, i.e., low characteristic path length and high clustering coefficient (Sporns et al., 2004). It has been seen that “synchronization” (as a collective dynamical behavior) occurs more rapidly in networks with small-world structure (Watts and Strogatz, 1998). Hence, we are able to use structural information (i.e., the pattern of connectivity between elements of the system) for understanding the functional pattern of the organization. Moreover, it is suggested that synchronization is the main mechanism for information exchange between different brain regions (Womelsdorf et al., 2007).

One type of abnormal synchronization between brain areas occurs during epileptic seizures which may lead to consciousness disturbance (Lehnertz et al., 2009). The synchrony in epileptic seizure is usually initiated in one special part of the brain and propagated to wide areas of it. Although the mechanism of this spread is not yet clear, preventing this spread will help to control the seizure. Although different pharmacological routes exist for controlling the seizures, it seems that more effective methods are needed to be introduced.

Previously Zhang et al. (2011) showed the usefulness of network theory approach in studying epilepsy. We hypothesize that applying the concept of “complex network controllability” to the brain could help in managing the epileptic seizures.

In order to have controllability, for each possible state (S) of network, a set of inputs (U) must be set up so that exerting it to all (or some) nodes force the network to a specific state (S1) (Liu et al., 2011). Liu et al. named the minimal subset of nodes which are able to control the network as “driver nodes: ND.” This Idea is utilized in different applications of networks such as genetic regulatory and wireless communication networks (Abdallah, 2011; Khan and Doostmohammadian, 2011).

Based on our hypothesis, controllability idea may help in managing the behavior of brain as a biological organization. For applying the concept of “complex network controllability” to the brain networks, it is necessary to find its driver nodes by using connectivity information, i.e., structural organization of the brain. Different neuro-imaging techniques such as Diffusion Tensor Imaging (DTI) provide a variety of information on brain connectivity networks (Le Bihan et al., 2001). DTI derived directed graphs are becoming increasingly available, also from clinical environments.

In a directed network, driver nodes could be found based on connectivity graph of the network. For this purpose, the maximum matching problem should be solved. Based on definition, maximum matching is the maximum set of links that don’t have the same start or end nodes. There are some search algorithms for finding the best maximum matching solution. A “matched node” is a node to which a link in the maximum matching points. Driver nodes are those nodes which are not matched (also named unmatched) (Liu et al., 2011). Therefore, for applying our hypothesis in a brain network connectivity graph, the maximum matching problem should be solved; then, If each driver node is directly controlled by external inputs (such as TMS, TDCS, and trans-cranial US), the whole brain network would be fully controlled and forced to the desired state.

During epileptic seizure, synchronization spread to wide areas of brain network is an undesired state. Exerting proper inputs (which could be via some stimulation methods) to driver nodes may prevent this spreading and force the network to the desired situation. Stimulation approaches so far have limited success (Conte et al., 2007). The stimulation site is mostly taken assuming some “focal” generator but it is increasingly becoming accepted that widespread networks might be underlying the focal seizures (Spencer, 2002). The work by Liu et al suggests a way to reduce the number of stimulation sites to be tested in animal experiments.

For evaluating our hypothesis, we suggest using some brain stimulation methods in order to excite or inhibit the driver nodes and prevent the spread of synchrony. For this purpose, different methods such as Trans-cranial Magnetic Stimulation (TMS), Trans-cranial Direct Current Stimulation (TDCS), and trans-cranial Ultrasound (US) stimulation may be used (Hallett, 2000; Nitsche and Paulus, 2000; Tufail et al., 2011). Surely, it is necessary to test our hypothesis first in animal models.

One empirical problem in this regard is that we do not know the exact time of seizure initiation. For solving this problem, data analysis methods may be used to predict the most probable ictal time.

In a recent study, targeting some neurons with a closed-loop optogenetic strategy and reducing their activity in real-time caused an immediate interrupt in seizures (Paz et al., 2013). We think that finding the driver nodes and targeting them systematically with this method may be more effective.

Epilepsy as one of the most common brain diseases involves the synchronization of wide spread areas of the brain. Managing epileptic seizures needs to suppress the spread of this undesired synchronization. Our hypothesis claims that applying the “complex networks controllability” approach to brain may enhance the management of seizure. For this purpose, the driver nodes of the brain should be found and then proper inputs must be exerted to them in order to force the state of the brain to normal conditions.

In an overview, brain is a biological organization. Based on our hypothesis, applying complex network approach to it may help in managing its abnormal higher level functions (states). In this regard, high level functions are an emergent property of cooperation between its elements in lower level of hierarchy. Therefore we suggest that in the brain, applying controllability idea (driving the final state of a complex system to a desired one), may help in managing its abnormal states via driving them to normal ones. Accordingly, there are some elements (nodes) which have a specific effect on the final state of the system.

Previous studies have suggested that nodes with more connections (hubs) are the most important ones in the sense of network information exchange. However, based on controllability idea, driver nodes are not necessarily hubs (Tang et al., 2012). In this regard, testing the relative importance of driver nodes and hubs in different brain diseases such as epilepsy needs to be evaluated experimentally.
